# Supplementary material for: Occurrence of Seahorses Hippocampus spp. in the Southernmost Part of Western Europe: A New Maximum Depth Record
Source: Animals (Basel). 2024 Aug 12;14(16):2328. doi: 10.3390/ani14162328 (PMC11350901; doi:10.3390/ani14162328)
Supplement: Supplementary file 1 [file animals-14-02328-s001.zip › animals-3113809-supplementary.pdf]

Occurrence of seahorses *Hippocampus spp.* in the southernmost part of Western Europe and new maximum depth record

Ignacio Ruiz-Jarabo, I., J. Hernández-Urcera, S. Pereira, I. Sobrino, J. A. López and M. Planas

Supplementary Table S1. Seahorse specimens captured during bottom-trawling oceanographic campaigns conducted in the Gulf of Cadiz (Spain) between October 1992 and October 2023 by the Spanish Institute of Oceanography (IEO-CSIC) as part of the *ARSA* surveys.

Hippocampus hippocampus			***Hippocampus guttulatus***		
**Specimens**	**Date**	**Depth (m)**	**Specimens**	**Date**	**Depth (m)**
2	10/10/1992	18	1	06/03/2009	92
2	18/03/1995	18	1	11/03/2011	26
2	18/03/1995	20	2	01/11/2020	64
1	03/03/1998	71			
2	03/12/2002	21			
1	01/11/2004	22			
1	19/03/2006	60			
1	12/11/2006	43			
1	15/03/2008	23			
2	20/03/2008	29			
1	05/03/2010	78			
1	19/03/2012	45			
1	23/03/2012	29			
1	24/03/2012	70			
1	13/11/2013	24			
1	29/10/2014	28			
1	02/03/2015	79			
1	06/11/2015	44			
1	06/11/2015	101			
1	31/10/2016	29			
1	31/10/2016	23			
3	31/10/2016	22			
1	05/11/2016	42			
1	22/02/2017	26			
1	25/02/2017	45			
2	25/02/2017	95			
1	31/10/2017	19			
1	19/02/2018	52			
2	19/02/2018	49			
1	25/02/2018	28			
1	04/11/2018	80			
1	01/11/2020	64			
2	11/03/2023	42			
2	11/03/2023	59			
1	12/03/2023	63			
2	30/10/2023	29			

Supplementary Table S2. Seahorse specimens reported in the Gulf of Cadiz and surrounding areas by artisanal fishermen (shown as "Artisanal") and other sources. * dead specimens located on the beach. ODM: Seawatchers (Observadores del Mar). FAMM: Fundación Aula del Mar Mediterráneo. [1] and [2]: personal communications by C.B. de los Santos and C. Morales-Caselles, respectively.

Species	Source	Specimens	Date	Sex	GBIF/ODM ID
H. hippocampus	Artisanal	1	15/07/2022		
	Artisanal	1	11/04/2023		
	Artisanal	1	18/05/2023		
	Artisanal	1	26/05/2023		
	Artisanal	1	19/06/2023		
	Artisanal	1	12/07/2023		
	iNaturalist	1	01/07/2012		diveboard:24141_206586_0
	iNaturalist	1	08/07/2012		diveboard:21101_206586_0
	iNaturalist	1	13/12/2019	Female	115439538
	iNaturalist	1	25/08/2020		126687636
	iNaturalist*	1	01/02/2022		236213724
	iNaturalist*	1	09/02/2022		236213653
	iNaturalist*	1	10/02/2022		236406710
	iNaturalist	1	31/05/2022	Juvenile	358340883
	iNaturalist	1	06/08/2022		287033704
	iNaturalist	1	13/10/2022	Male	308827029
	iNaturalist	1	02/07/2023		381642506
	iNaturalist*	1	27/07/2023		392002828
	iNaturalist	1	17/08/2023		422764289
	iNaturalist*	1	01/02/2024		199491922
	OdM	1	13/08/2023	Male	22626
	OdM	1	20/08/2023	Male	22628
	OdM	1	23/09/2023	Female	22787
	Pers. Com. [1]	1	01/03/2011	Male	
	Pers. Com. [2]*	1	02/06/2022	Female	
H. guttulatus	iNaturalist	1	20/06/2013		4217716
	iNaturalist	1	09/02/2020	Male	86894933
	iNaturalist	1	28/06/2020	Male	113737393
	iNaturalist	1	01/07/2020	Female	115439190
	iNaturalist	1	01/07/2020	Male	114262381
	iNaturalist	1	01/07/2020		114261796
	iNaturalist	1	25/08/2020	Female	126687146
	iNaturalist	1	25/08/2020	Male	126686223
	iNaturalist	1	06/08/2022		287033145
	iNaturalist	1	20/08/2022		290647650
	iNaturalist	1	27/08/2022		292366171
	iNaturalist	1	09/09/2022		295747505
	iNaturalist	1	27/11/2022	Male	316925141
	iNaturalist	1	29/01/2023	Male	330792331
	iNaturalist	1	12/03/2023	Male	341126473
	iNaturalist	1	21/05/2023		366243984
	iNaturalist	1	02/07/2023	Male	381643119
	iNaturalist	1	02/07/2023	Female	381643979

iNaturalist	1	21/07/2023		388445449
iNaturalist	1	14/01/2024		448164776
ODM	1	13/08/2023	Male	22619
ODM	1	20/08/2023	Male	22627
ODM	1	20/08/2023	Male	22629
ODM	1	23/09/2023		22786
ODM	1	08/10/2023	Female	22876
FAMM	4	21/08/2023		
FAMM	6	02/09/2022		
FAMM	7	08/09/2022		
